# An ω-3, but Not an ω-6 Polyunsaturated Fatty Acid Decreases Membrane Dipole Potential and Stimulates Endo-Lysosomal Escape of Penetratin

**DOI:** 10.3389/fcell.2021.647300

**Published:** 2021-04-12

**Authors:** Florina Zakany, Mate Szabo, Gyula Batta, Levente Kárpáti, István M. Mándity, Péter Fülöp, Zoltan Varga, Gyorgy Panyi, Peter Nagy, Tamas Kovacs

**Affiliations:** ^1^Division of Biophysics, Department of Biophysics and Cell Biology, Faculty of Medicine, University of Debrecen, Debrecen, Hungary; ^2^Department of Genetics and Applied Microbiology, Faculty of Science and Technology, University of Debrecen, Debrecen, Hungary; ^3^Department of Organic Chemistry, Faculty of Pharmacy, Semmelweis University, Budapest, Hungary; ^4^Lendület-Artificial Chloride Ion Transporter Group, Institute of Materials and Environmental Chemistry, Research Center for Natural Sciences, Budapest, Hungary; ^5^Division of Metabolism, Department of Internal Medicine, Faculty of Medicine, University of Debrecen, Debrecen, Hungary

**Keywords:** membrane dipole potential, polyunsaturated fatty acids, cholesterol, penetratin, flow cytometry

## Abstract

Although the largely positive intramembrane dipole potential (DP) may substantially influence the function of transmembrane proteins, its investigation is deeply hampered by the lack of measurement techniques suitable for high-throughput examination of living cells. Here, we describe a novel emission ratiometric flow cytometry method based on F66, a 3-hydroxiflavon derivative, and demonstrate that 6-ketocholestanol, cholesterol and 7-dehydrocholesterol, saturated stearic acid (SA) and ω-6 γ-linolenic acid (GLA) increase, while ω-3 α-linolenic acid (ALA) decreases the DP. These changes do not correlate with alterations in cell viability or membrane fluidity. Pretreatment with ALA counteracts, while SA or GLA enhances cholesterol-induced DP elevations. Furthermore, ALA (but not SA or GLA) increases endo-lysosomal escape of penetratin, a cell-penetrating peptide. In summary, we have developed a novel method to measure DP in large quantities of individual living cells and propose ALA as a physiological DP lowering agent facilitating cytoplasmic entry of penetratin.

## Introduction

An ever-increasing amount of evidence supports the active role of the cell membrane, and its lipid components in particular, in the regulation of the structure and function of transmembrane proteins and consequently a wide array of cellular functions. In general, lipids are thought to influence proteins through direct ligand-like interactions and/or indirect mechanisms that include changes in bulk membrane biophysical parameters (Corradi et al., 2019; Zakany et al., 2020). Among the latter, dipole potential (DP) is the most enigmatic factor, which originates from the non-random alignment of dipolar segments of carbonyl groups, cholesterol and membrane-associated water molecules. This preferential arrangement results in the generation of a large positive intramembrane electrostatic potential with an estimated magnitude of 150–450 mV, which is associated with a dipole electric field much stronger than that of the transmembrane or surface potentials (10^8^–10^9^ vs. 2.5 × 10^7^ and 10^6^ V/m, respectively) (O’Shea, 2003, 2005; Wang, 2012). The most important determinant of the magnitude of DP is the lipid composition of the membrane, i.e., the chemical types and amounts of phospholipids (Starke-Peterkovic and Clarke, 2009) and sterols (Simon et al., 1992; Haldar et al., 2012; Sarkar et al., 2017). Consistently, we have recently shown that the magnitude of DP shows lateral heterogeneity with higher values in lipid raft microdomains in the cell membrane (Kovacs et al., 2017). Experimentally, DP is most commonly increased by incorporating a sterol derivative, 6-ketocholestanol (6KC), into the membrane, which results in large alterations in the value of DP (Gross et al., 1994; Clarke and Kane, 1997; Kovacs et al., 2016, 2017). On the other hand, it is usually lowered experimentally with phloretin, however, the magnitude of changes induced by this natural phenol is relatively low (Gross et al., 1994; Clarke and Kane, 1997; Kovacs et al., 2016, 2017). Although reduction in cellular cholesterol levels by statin treatment is capable of reducing the DP (Sarkar et al., 2017; Batta et al., 2020), an efficient and non-pharmacological way of lowering DP has not been described yet. ω-3 and ω-6 polyunsaturated fatty acids, such as α-linolenic acid (ALA) (and its derivatives eicosapentaenoic acid (EPA) or docosahexaenoic acid (DHA)) and γ-linolenic acid (GLA) [and its derivative arachidonic acid (AA)] generally exert opposing effects on membrane biophysical parameters when compared to sterols. These include increased membrane fluidity (Calder et al., 1994; Leifert et al., 1999; Rajamoorthi et al., 2005), higher degree of hydration (Huster et al., 1997), decreased thickness and increased bending elasticity of lipid bilayers (Rawicz et al., 2000; Rajamoorthi et al., 2005). Consistent with these effects, it can be assumed that polyunsaturated fatty acids (PUFAs) might influence the magnitude of DP as well, however, to our knowledge, this hypothesis has not been examined in living cells yet.

Due to the mostly non-uniform charge distribution of proteins, the immense DP-associated electric field can be essential in the regulation of the conformational stability and consequently the functional activity of membrane proteins (O’Shea, 2003; Richens et al., 2015; Zakany et al., 2020). Consistently, DP was suggested to influence the function of bacterial ionophores (Ostroumova et al., 2012), voltage-gated ion channels (Pearlstein et al., 2017; Zakany et al., 2019), Na^+^/K^+^ ATPase (Clarke, 2015), P-glycoprotein (Davis et al., 2015) serotonin receptors (Bandari et al., 2014) and ErbB proteins (Kovacs et al., 2016). The DP can also modify membrane binding of drugs (Asawakarn et al., 2001), β-amyloid (Hertel et al., 1997) and other peptides (Cladera and O’Shea, 1998; Zhan and Lazaridis, 2012). Cell-penetrating peptides are promising therapeutic tools for the non-toxic delivery of cell-impermeable agents (Guidotti et al., 2017), however, their applicability is limited by low bioavailability (Wang et al., 2014). These peptides can enter the cytoplasm, i.e., the site of their action, through direct plasma membrane translocation or endocytosis followed by endo-lysosomal release (Futaki, 2006; Guidotti et al., 2017). Since both mechanisms involve interactions with the membrane, the DP-associated electric field might influence their cellular uptake, especially in the cases of charged cell-penetrating peptides, such as the cationic penetratin. Consistently, we have shown recently that decreases in DP in response to phloretin or atorvastatin-induced reduced cholesterol levels result in increased cellular uptake and endo-lysosomal escape of penetratin (Batta et al., 2020).

Despite its presumable biological relevance, studies examining DP are scarcely documented mainly due to difficulties in its quantification. The applicability of most DP measurement techniques, including cryoelectron microscopy (Wang et al., 2006), molecular dynamics simulations (Harder et al., 2009; Ding et al., 2015), atomic force microscopy (Yang et al., 2008) and vibrational Stark effect spectroscopy (Shrestha et al., 2017), is limited for the examination of living cells. Investigation of DP in living cells is mainly carried out with di-8-ANEPPS, an electrochromic dye using an excitation ratiometric assay in spectrofluorometry (Gross et al., 1994; Clarke and Kane, 1997) or microscopy (Kovacs et al., 2016, 2017). Alternatively, certain 3-hydroxyflavone derivatives, such as F66 can be used via an emission ratiometric assay due to their excited-state intramolecular proton transfer (ESIPT) reaction resulting in normal (N^∗^) and tautomer (T^∗^) excited states with well-separated bands in their emission spectra. This reaction is modulated by the strength of the local electric field, thus, the magnitude of DP. Consistently, these dyes were used to examine DP in spectrofluorometric (Klymchenko et al., 2003; Shynkar et al., 2005; Darwich et al., 2013) and microscopic assays (Shynkar et al., 2005; Darwich et al., 2013; Kovacs et al., 2017). While fluorescence microscopy provides single cell resolution with low throughput, spectrophotometry can measure a large number of cells without discrimination between live and dead cells. Flow cytometry combines the advantages of the two techniques, i.e., to measure DP in large quantities of individual living cells, however, to our knowledge, no such method has been published.

Here, we report on a novel flow cytometry method for the measurement of DP in individual living cells using F66, a 3-hydroxiflavon derivative and an emission ratiometric assay. Using this new technique, we show that sterols, stearic acid or γ-linolenic acid dose-dependently increased, while α-linolenic acid decreased DP, which did not result from changes induced in cell viability or membrane fluidity. Alterations in DP showed good correlation with changes in membrane hydration. Modifications in DP in response to fatty acids strongly correlated with their effects on endo-lysosomal escape of penetratin. Since membrane crossing of penetratin has been shown to correlate with the DP (Batta et al., 2020), these results confirm the biological relevance of our findings. Furthermore, we identified α-linolenic acid as a physiological DP lowering agent, which can be used in future studies examining the biological importance of DP.

## Results

### Effects of Phloretin and 6-Ketocholestanol on the Magnitude of Membrane Dipole Potential

**FIGURE 1:**
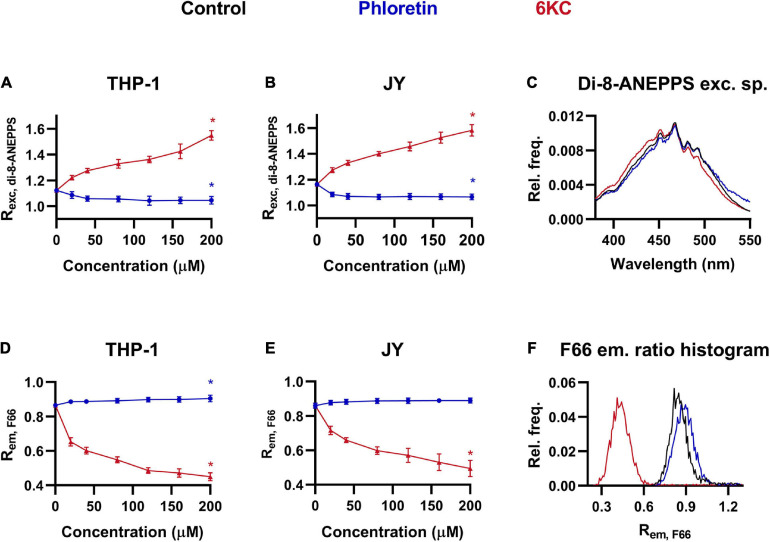
Effects of phloretin and 6-ketocholestanol (6KC) on the magnitude of dipole potential. THP-1 **(A)** or JY cells **(B)** were treated with phloretin or 6KC at various concentrations between 20 and 200 μM and labeled with di-8-ANEPPS followed by determination of the dipole potential-sensitive excitation ratio (R_*exc, di–8–ANEPPS*_) using spectrofluorometry. R_*exc, di–8–ANEPPS*_ values were calculated from fluorescence intensities integrated between excitation wavelengths 410–440 nm and 490–520 nm and the mean (± SD) values of five independent experiments were plotted as a function of the applied concentrations of phloretin (dark blue) and 6KC (red). Representative excitation spectra display shifts induced by phloretin and 6KC compared to controls (black) **(C)**. Alternatively, control THP-1 **(D)** or JY **(E)** cells and those treated with phloretin or 6KC were labeled with F66 and the emitted fluorescence intensities of individual cells were measured in the wavelength ranges 470–490 nm and 564–606 nm and the mean intensity ratio (R_*em, F66*_) was calculated from data of at least 20,000 living cells per sample. The average values of five independent measurements (± SD) were plotted as a function of the applied concentrations of phloretin and 6KC. Representative histograms containing data of individual cells display the shifts in R_*em*, *F66*_ values in response to phloretin and 6KC compared to controls **(F)**. Asterisks (*) indicate significant differences obtained at maximal treatment concentrations compared to control samples (*p* < 0.05, ANOVA followed by Tukey’s HSD test).

In order to provide a reference for a new, flow cytometry method for measuring DP, we employed a widely used voltage-sensitive fluorophore, di-8-ANEPPS in spectrofluorometry to follow changes in DP in response to the most commonly applied DP modifying agents, phloretin and 6-ketocholestanol (6KC), which were incorporated into the cell membrane with the help of Pluronic-F-127, a hydrophilic non-ionic surfactant (Gross et al., 1994; Clarke and Kane, 1997; Kovacs et al., 2016, 2017). Consistent with previous reports, 6KC dose-dependently increased the ratio of fluorescence intensities integrated in the blue edge (410–440 nm) and the red edge (490–520 nm) of the excitation spectrum of the dye (R_*exc, di–8–ANEPPS*_) positively correlating with the magnitude of DP in two model cell lines, THP-1 and JY (Figures 1A,B). Maximal changes were observed at the maximal examined dose of 200 μM. On the other hand, phloretin dose-dependently decreased the excitation ratio, however, changes were rather modest (but statistically significant) even at the maximal applied concentration of 200 μM. The blue-shift of di-8-ANEPPS excitation spectrum in response to 6KC and its slight red-shift after phloretin were also demonstrated by representative spectra shown in Figure 1C.

Since certain 3-hydroxiflavon derivatives exhibit a spectral change in their emission in response to alterations in the intramembrane electric field (Klymchenko et al., 2003; Shynkar et al., 2005; Darwich et al., 2013; Kovacs et al., 2017), these fluorophores are theoretically suitable for flow cytometric examination of the magnitude of DP. To test this hypothesis, we repeated our experiments with THP-1 and JY cells treated with different concentrations of phloretin and 6KC using flow cytometry and F66, a 3-hydroxiflavon fluorophore, and determined the ratio of fluorescence intensities of the dye (R_*em, F66*_) corresponding to its normal (N^∗^) and tautomeric (T^∗^) excited form measured in the ranges of 470–490 nm and 564–606 nm, respectively, which negatively correlates with the magnitude of DP (Darwich et al., 2013; Kovacs et al., 2017). In good keeping with our results obtained with di-8-ANEPPS, 6KC resulted in dose-dependent decreases in the R_*em, F66*_ (Figures 1D,E), referring to increased DP. Although phloretin led to increased R_*em, F66*_ values implying lower magnitudes of DP, this change did not reach the level of significance in one of the cell lines. The alterations induced by these treatments were also demonstrated by representative histograms based on R_*em, F66*_ values of individual cells, since the curves were shifted to lower and higher values in response to 6KC and phloretin, respectively (Figure 1F).

### Effects of Different Sterols on the Magnitude of Membrane Dipole Potential

**FIGURE 2:**
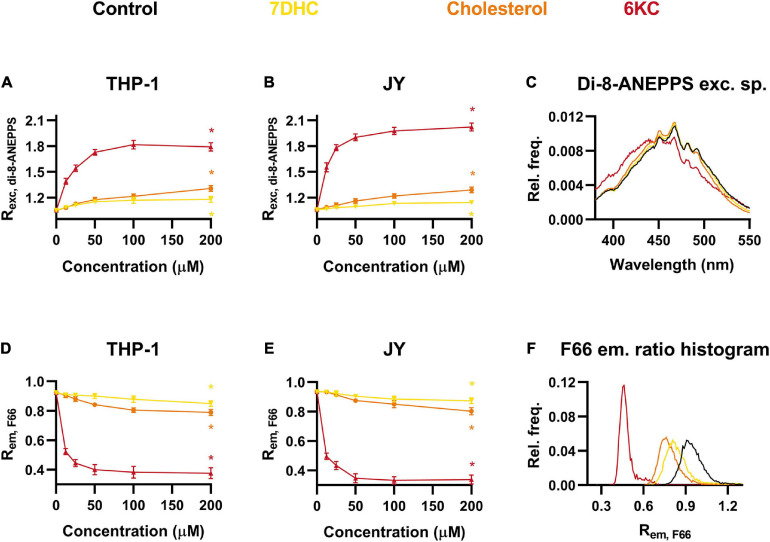
Effects of 7-dehydrocholesterol (7DHC), cholesterol and 6-ketocholestanol (6KC) on the magnitude of dipole potential. THP-1 **(A)** or JY cells **(B)** were treated with 7DHC, cholesterol or 6KC at various concentrations between 12.5 and 200 μM and labeled with di-8-ANEPPS, which was followed by determination of the dipole potential-sensitive excitation ratio (R_*exc, di–8–ANEPPS*_) using spectrofluorometry. R_*exc, di–8–ANEPPS*_ values were calculated from fluorescence intensities integrated between excitation wavelengths 410–440 nm and 490–520 nm and the mean (± SD) values of five independent experiments were plotted as a function of the applied concentrations of 7DHC (yellow), cholesterol (orange) and 6KC (red). Representative excitation spectra display shifts induced by the various sterols compared to controls (black) **(C)**. Alternatively, control THP-1 **(D)** or JY **(E)** cells and those treated with 7DHC, cholesterol or 6KC were labeled with F66 and the emitted fluorescence intensities of individual cells were measured in the wavelength ranges 470–490 nm and 564–606 nm and the mean intensity ratio (R_*em, F66*_) was calculated from data of at least 20,000 living cells per sample. The average values of five independent measurements (± SD) were plotted as a function of the applied concentrations of 7DHC, cholesterol and 6KC. Representative histograms containing data of individual cells display the shifts in R_*em, F66*_ values in response to the different sterols compared to controls **(F)**. Asterisks (*) indicate significant differences obtained at maximal treatment concentrations compared to control samples (*p* < 0.05, ANOVA followed by Tukey’s HSD test).

Since the magnitude of DP is very efficiently modulated by the sterol content of the membrane (Simon et al., 1992; Haldar et al., 2012; Sarkar et al., 2017), we validated our novel method using cells exogenously treated with different sterols including 7-dehydrocholesterol (7DHC), cholesterol and 6KC complexed with methyl-beta-cyclodextrin (MβCD). In these experiments, 6KC was used in complex with MβCD to ensure the comparability of the effects of different sterols. It was shown previously that all of them are capable of elevating DP with 6KC being the most efficient and 7DHC being the weakest (Simon et al., 1992; Smondyrev and Berkowitz, 2001; Starke-Peterkovic et al., 2006; Haldar et al., 2012). Consistent with these findings, we observed with spectrofluorometry that all of the sterols resulted in significantly increased R_*exc, di–8–ANEPPS*_ values, i.e., elevated DP levels, in a dose-dependent manner. At the maximal applied concentration of 200 μM, R_*exc, di–8–ANEPPS*_ was slightly raised after 7DHC, moderately increased by cholesterol and robustly elevated in response to 6KC in both THP-1 and JY cells (Figures 2A,B). All of these changes were found statistically significant. The blue-shifts of different degrees induced by sterols were also obviously visible in the excitation spectra of di-8-ANEPPS in response to these compounds (Figure 2C).

The experiments repeated using F66-stained THP-1 cells in flow cytometry showed that R_*em, F66*_ of control cells was significantly and dose-dependently decreased by all of the three examined sterols in magnitudes correlating perfectly with those observed with di-8-ANEPPS (Figure 2D). Alterations of R_*em, F66*_ in F66-labeled JY cells were similar to those measured in THP-1 cells and correlated with the results obtained with di-8-ANEPPS staining (Figure 2E). All of these changes were found statistically significant. These alterations were also obvious in R_*em, F66*_ histograms of individual cells, as sterols shifted the curve to lower values to a different extent (Figure 2F).

### Effects of Different Fatty Acids on the Magnitude of Dipole Potential

**FIGURE 3:**
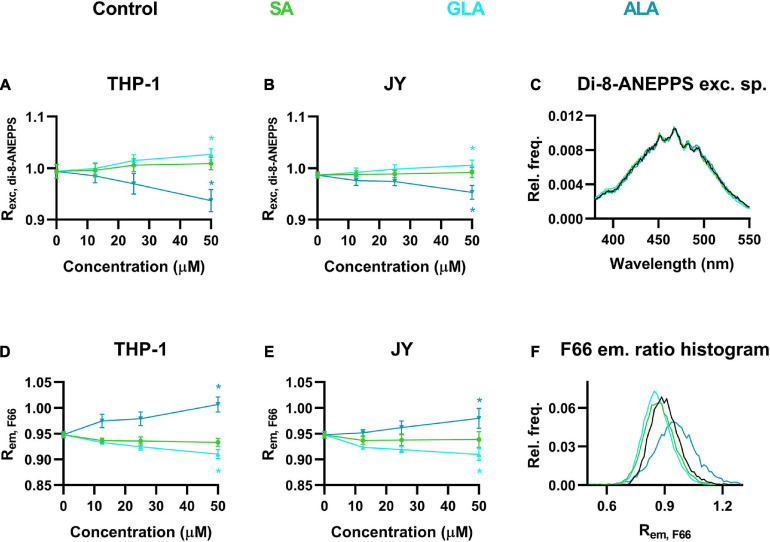
Effects of α-linolenic acid (ALA), γ-linolenic acid (GLA) and stearic acid (SA) on the magnitude of dipole potential. THP-1 **(A)** or JY cells **(B)** were treated with ω-3 ALA, ω-6 GLA or fully saturated SA at various concentrations ranging from 12.5 to 50 μM and labeled with di-8-ANEPPS, which was followed by determination of the dipole potential-sensitive excitation ratio (R_*exc, di–8–ANEPPS*_) using spectrofluorometry. R_*exc, di–8–ANEPPS*_ values were calculated from fluorescence intensities integrated between excitation wavelengths 410–440 nm and 490–520 nm and the mean (± SD) values of five independent experiments were plotted as a function of the applied concentrations of ALA (blue), GLA (cyan) and SA (green). Representative excitation spectra display shifts induced by the various fatty acids compared to controls (black) **(C)**. Alternatively, control THP-1 **(D)** or JY **(E)** cells and those treated with ALA, GLA or SA were labeled with F66 and the emitted fluorescence intensities of individual cells were measured in the wavelength ranges 470–490 nm and 564–606 nm and the mean intensity ratio (R_*em, F66*_) was calculated from data of at least 20,000 living cells per sample. The average values of five independent measurements (± SD) were plotted as a function of the applied concentrations of ALA, GLA, and SA. Representative histograms containing data of individual cells display the shifts in R_*em, F66*_ values in response to the different fatty acids compared to controls **(F)**. Asterisks (*) indicate significant differences obtained at maximal treatment concentrations compared to control samples (*p* < 0.05, ANOVA followed by Tukey’s HSD test).

Although sterols are thought to be the most important determinants of DP, other lipids might also influence its magnitude (Starke-Peterkovic and Clarke, 2009). Therefore, we next examined the effects of various fatty acids including the polyunsaturated ω-3 α-linolenic acid (ALA) and ω-6 γ-linolenic acid (GLA), and their fully saturated counterpart, stearic acid (SA) at different concentrations. When cells were incubated in the presence of 12.5 to 50 μM of these fatty acids for 48 h, we observed dose-dependent changes in the magnitude of DP. Remarkably, ALA lowered DP, as evidenced by the significantly decreased R_*exc, di–8–ANEPPS*_ values obtained in response to the maximal applied concentration of 50 μM in both THP-1 and JY cells (Figures 3A,B). On the other hand, SA resulted in slightly higher R_*exc, di–8–ANEPPS*_ values that might refer to an elevated DP, however, these changes did not reach the level of significance. To our surprise, GLA induced significant increases in R_*exc, di–8–ANEPPS*_ showing higher DP (Figures 3A,B). These relatively slight changes in response to fatty acids were visible in di-8-ANEPPS excitation spectra as well (Figure 3C).

We tested the effects of these fatty acids using our novel flow cytometry technique as well. Strongly consistent results were found further corroborating the applicability of our method. In THP-1 cells, R_*em, F66*_ of untreated cells was dose-dependently and significantly increased by ALA referring to lowered DP, while opposite changes were observed in response to SA or GLA with the former not reaching the level of significance (Figure 3D). The fatty acid-induced alterations in F66-labeled JY cells coincided in their magnitude and statistical significance with those obtained with THP-1 cells (Figure 3E). Shifts in R_*em, F66*_ values were demonstrated by representative histograms showing cell-by-cell data as well (Figure 3F).

### Effects of Different Sterols and Fatty Acids on Cell Viability, Membrane Fluidity and Membrane Hydration

**FIGURE 4:**
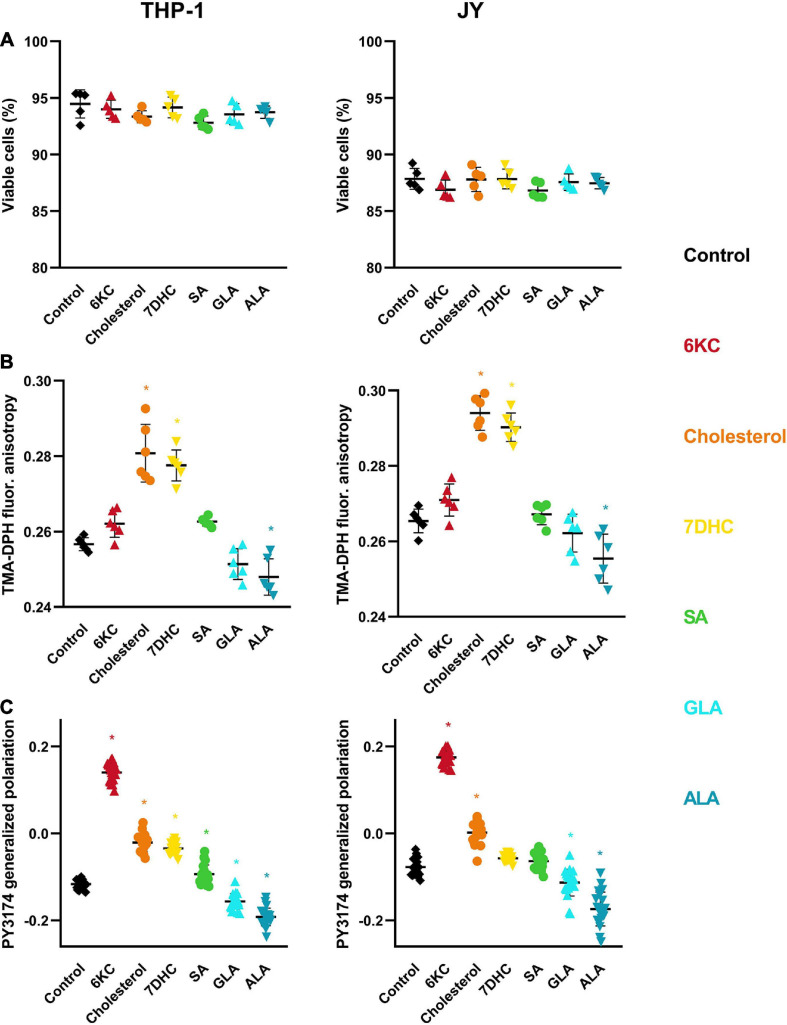
Effects of sterols and fatty acids on cell viability, membrane fluidity and hydration. **(A)** THP-1 or JY cells were treated with 200 μM 7DHC (yellow), cholesterol (orange) or 6KC (red) for 1 h, or 50 μM ALA (blue), GLA (cyan) or SA (green) for 48 h, and subsequently labeled with the necrosis marker Sytox Green and the apoptosis marker AlexaFluor647-conjugated annexin V. Fluorescence intensities of individual cells were measured using a flow cytometer and the fraction of Sytox Green and annexin V negative living cells was calculated for each sample containing at least 20,000 cells. Fractions of living cells obtained in five independent experiments and their average values (± SD) are plotted for the different treatments. **(B)** THP-1 and JY cells treated as above were labeled with TMA-DPH and the fluorescence anisotropy of the fluorophore was determined with spectrofluorometry. Anisotropy values obtained in six independent experiments and their average values (± SD) are plotted for the different treatments. **(C)** THP-1 and JY cells treated as above were labeled with PY3174 and the generalized polarization of the dye localized in the cell membrane was determined using confocal microscopy and quantitative image analysis. Mean generalized polarization values of 20 individual images obtained in five independent experiments and their average values (± SD) are plotted for the different treatments. Each image contained data of 10–15 cells of normal morphology with a total number of 200–300 cells per treatment. Asterisks (*) indicate significant differences compared to control samples (*p* < 0.05, ANOVA followed by Tukey’s HSD test).

Environment-sensitive fluorophores, such as F66, can be influenced by membrane-related parameters other than DP. For example, spectral properties of F66 were previously shown to be largely altered when cells undergo apoptosis (Darwich et al., 2013). To rule out the possibility that the observed changes were modified by alterations in the viability of cells, cells treated with the different lipids were labeled with the necrosis marker Sytox Green and the apoptosis marker AlexaFluor 647-conjugated annexin V. We observed no significant changes in the fraction of double negative, viable cells in response to treatments even at the maximal concentrations applied in any of the two examined cell types (Figure 4A).

Next, we tested if the observed changes in R_*em, F66*_ were related to alterations in membrane fluidity. We labeled control and treated cells with TMA-DPH, and determined its fluorescence anisotropy using spectrofluorometry, which inversely correlates with membrane fluidity (Batta et al., 2018). In both cell lines, the anisotropy of controls was significantly elevated by 200 μM of cholesterol and 7DHC, whereas the effect of 6KC did not reach statistical significance (Figure 4B). This efficacy order was different from their effect on the DP. From among the fatty acids, ALA significantly decreased TMA-DPH anisotropy, while the effect of SA and GLA did not reach statistical significance.

Since one of the major origins of DP is the arrangement of membrane-associated water molecules, we tested if membrane hydration is changed in response to treatments with sterols or fatty acids. We labeled control and treated cells with PY3174, a fluorophore related to the widely used Laurdan, followed by confocal microscopy and quantitative image analysis to determine the average value of generalized polarization from data corresponding to cell membrane pixels. Generalized polarization of the dye was previously shown to correlate with the hydrophobicity of its environment, i.e., it inversely correlates with the degree of membrane hydration (Kwiatek et al., 2013). In both cell lines, the generalized polarization of controls was shifted toward the positive direction in the following efficacy order: 6KC > cholesterol ≥ 7DHC (Figure 4C). Furthermore, while SA also increased the generalized polarization, albeit to a miniscule extent, both GLA and ALA reduced it implying increased membrane hydration. All of these alterations were found statistically significant except for the effects of 7DHC and SA in JY cells.

### Correlation Between Changes in Dipole Potential Revealed by Flow Cytometry and Spectrofluorometry, and Alterations Induced in Membrane Fluidity or Membrane Hydration

**FIGURE 5:**
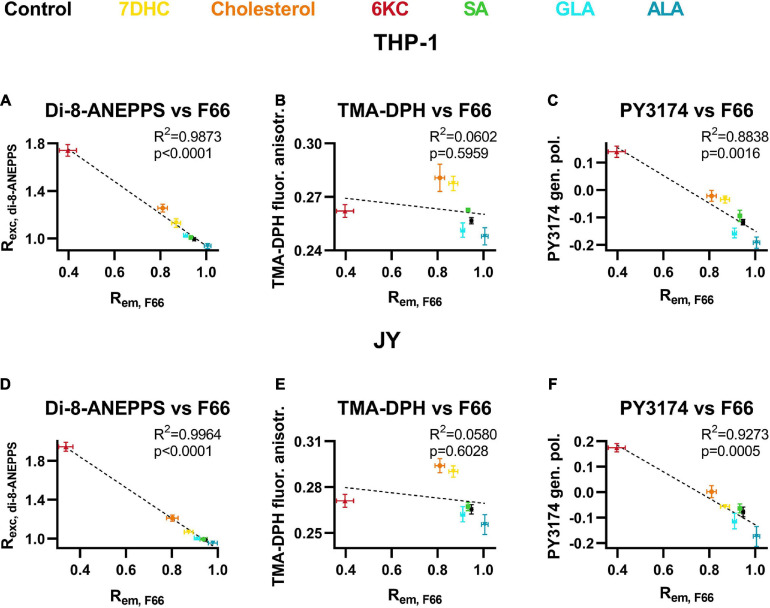
Correlations between F66 emission ratio and di-8-ANEPPS excitation ratio, TMA-DPH fluorescence anisotropy and PY3174 generalized polarization. THP-1 **(A–C)** or JY **(D–F)** cells were treated with 200 μM 7DHC (yellow), cholesterol (orange) or 6KC (red) for 1 h, or 50 μM ALA (blue), GLA (cyan) or SA (green) for 48 h, which was followed by labeling and determination of F66 emission ratio, di-8-ANEPPS excitation ratio, TMA-DPH fluorescence anisotropy and PY3174 generalized polarization, as described previously in detail. Average values (± SD) of the F66 excitation ratio were plotted as function of di-8-ANEPPS excitation ratios **(A,D)**, TMA-DPH fluorescence anisotropy **(B,E)** or PY3174 generalized polarization values **(C,F)**. R^2^ and *p* values determined with linear regression analysis are shown in the panels.

To further validate the applicability of the novel flow cytometry method described in this study, we compared our results obtained with F66 after treatments with the different sterols and fatty acids and those reported by reference measurements with di-8-ANEPPS quantitatively. R^2^ and *p* values determined during linear regression analysis showed excellent correlation between di-8-ANEPPS and F66 ratios in both cell types (Figures 5A,D) strongly supporting the utility of our novel approach for DP measurement. Then, we determined the correlation between F66 ratios and TMA-DPH fluorescence anisotropy values reporting membrane fluidity after lipid treatments. No significant correlation was found between the two parameters arguing against a large contribution of membrane fluidity to changes induced in F66 spectrum (Figures 5B,E). On the other hand, when comparing F66 ratios with PY3174 generalized polarization values, we found a significant positive correlation between the two implying strong association between the magnitude of DP and membrane hydration (Figures 5C,F).

### α-Linolenic Acid (But Not γ-Linolenic Acid or Stearic Acid) Counteracted Cholesterol-Induced Increases in Membrane Dipole Potential

**FIGURE 6:**
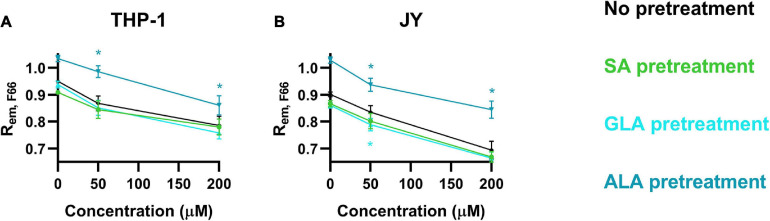
Combined effects of α-linolenic acid (ALA), γ-linolenic acid (GLA) and stearic acid (SA) on the magnitude of dipole potential. THP-1 **(A)** or JY cells **(B)** were pretreated with 50 μM ω-3 ALA, ω-6 GLA or fully saturated SA followed by treatment with 50 or 200 μM cholesterol, labeling with F66, measurement of the emitted fluorescence intensities of individual cells in the wavelength ranges 470–490 nm and 564–606 nm and calculation of the mean intensity ratio (R_*em, F66*_) from data of at least 20,000 living cells per sample. The average values of five independent measurements (± SD) were plotted as a function of the applied concentrations of cholesterol after pretreatment with ALA (blue), GLA (cyan) and SA (green). Asterisks (*) indicate significant differences compared to samples without pretreatment (*p* < 0.05, ANOVA followed by Tukey’s HSD test).

We next examined the combined effects of cholesterol and fatty acids on the magnitude of DP using the flow cytometric method described in the manuscript. We pretreated cells with 50 μM ALA, GLA or SA for 48 h followed by a 1 h incubation in the presence of 50 or 200 μM cholesterol. Treatment of cells with cholesterol or any of the fatty acids as a single agent resulted in DP changes similar to those described in the previous sections with cholesterol significantly increasing DP, while the DP-enhancing effect of GLA and SA, and the DP-reducing effect of ALA were less marked (Figures 5A,B). Pretreatment of either cell type with ALA significantly decreased the DP-elevating effect of cholesterol, while a tendency to augment the cholesterol-induced DP changes by SA and GLA was observed, although these changes usually did not reach the level of significance (Figures 6A,B).

### α-Linolenic Acid (But Not γ-Linolenic Acid or Stearic Acid) Resulted in Increased Endo-Lysosomal Escape of Penetratin

**FIGURE 7:**
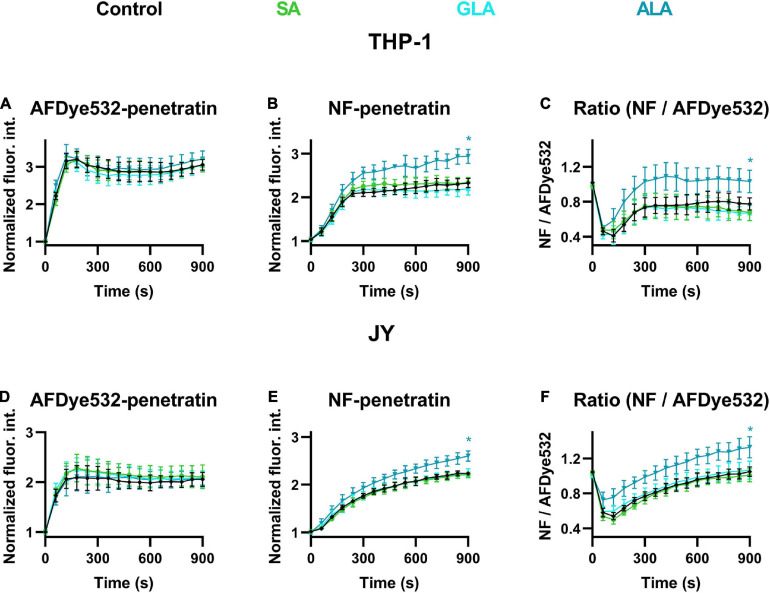
Effects of α-linolenic acid (ALA), γ-linolenic acid (GLA) and stearic acid (SA) on the cellular uptake and endo-lysosomal escape of penetratin. Control THP-1 **(A–C)** or JY cells **(D–F)** (black) or those treated with 50 μM ALA (blue), GLA (cyan) or SA (green) for 48 h were incubated at 37°C in the continuous presence of 5 μM of AFDye532- and 5 μM of NF-labeled penetratin. Fluorescence intensities of individual cells were measured using time-correlated flow cytometry. After gating out debris and non-viable cells, the average fluorescence intensities of AFDye532 **(A,D)**, NF **(B,E)** and their ratios **(C,F)** were calculated in 10 s-periods and normalized to minimal values using data from five independent experiments per sample. Every 6th data point (± SD) was plotted as a function of time. Asterisks (*) indicate significant differences compared to control samples at 900 s (*p* < 0.05, ANOVA followed by Tukey’s HSD test).

To test the biological relevance of alterations in the magnitude of DP by fatty acids, we next examined the effects of SA, GLA, and ALA on the cellular uptake and endo-lysosomal release of penetratin, a positively charged cell-penetrating peptide. We have recently shown that a reduced magnitude of DP resulting from phloretin treatment or cholesterol depletion by atorvastatin leads to significantly increased cytoplasmic entry of penetratin mainly through facilitation of its endo-lysosomal escape (Batta et al., 2020). Here, we treated cells with 50 μM ALA, GLA or SA for 48 h, then incubated them in the continuous presence of an equimolar mixture of AFDye532- and naphthofluorescein (NF)-labeled penetratin at 37°C and we measured the fluorescence intensities of individual living cells, excluding DAPI-positive ones, in a time-correlated manner. AFDye532 exhibits pH-independent fluorescence, thus its signal is proportional to the total cellular uptake of penetratin. On the contrary, the fluorescence of NF is quenched at acidic pH, therefore, its intensity characterizes the amount of penetratin in non-acidic compartments (mainly the cytosol). The ratio of NF and AFDye532 fluorescence signals gives information about the fractional escape of penetratin from acidic compartments, i.e., the endo-lysosomal system. When studying the kinetics of total cellular uptake and endo-lysosomal release of the cationic cell-penetrating peptide in THP-1 and JY cells, we observed results similar to those reported previously for other cell lines. The signal of AFDye532 corresponding to the total cellular penetratin concentration showed saturation at 120–180 s (Figures 7A,D), while the increase in NF signal referring to the endo-lysosomal escape of penetratin was significantly delayed with a fast increase in the first 180–240 s and a slower continuous rising phase afterward (Figures 7B,E). The ratio of NF and AFDye532 characterizing the fraction of penetratin in the cytosol initially declined due to the presence of penetratin in endo-lysosomes, which was followed by a continuous gradual increase (Figures 7C,F). When examining the effects of fatty acids on AFDye532 fluorescence, i.e., total cellular penetratin uptake, no changes were observed in normalized fluorescence intensities after SA, GLA or ALA compared to untreated samples. On the other hand, NF intensity was significantly elevated in response to ALA when compared to controls, while no significant differences were observed after SA or GLA. Consistent with the previous data, the ratio of NF and AFDye532 intensities was significantly increased by ALA, while SA or GLA exerted no significant changes when compared to controls. These data suggested that the endo-lysosomal release of penetratin was significantly enhanced by ALA (but not SA or GLA) in both THP-1 and JY cells without any significant alterations in its total cellular uptake.

These contrasting effects of ALA and GLA exerted on the endo-lysosomal penetratin escape might originate from (i) opposite alterations induced by these fatty acids in the magnitude of DP; or (ii) the fact that GLA treatment does not reach endo-lysosomal membranes. However, the latter was ruled out by confocal microscopy experiments demonstrating that both ALA and GLA decreased generalized polarization of PY3174 in the lysosomal membranes of THP-1 and JY cells, visualized by LysoTracker Deep Red, to similar extents as observed previously in the cell membrane (Supplementary Figure 1). These observations strongly argue against differences in ALA and GLA effects on penetratin escape resulting from substantial variations in their efficiencies to modify endo-lysosomal membranes.

## Discussion

Despite the logical assumption that the dipole potential (DP) can substantially mediate the indirect effects of lipids on transmembrane proteins, effects of DP on membrane proteins are scarcely documented (O’Shea, 2003, 2005; Wang, 2012; Zakany et al., 2020). Such studies are mainly hampered by the lack of (i) a method suitable for high-throughput measurement of DP in living cells; and (ii) a physiological and easy-to-use DP lowering agent. Our study was mainly motivated by these shortcomings and led to the following major conclusions: (i) flow cytometric detection of the spectral shift in the emission spectrum of F66 is suitable for sensitive, high-throughput measurement of DP in living cells; (ii) α-linolenic acid, an ω-3 polyunsaturated fatty acid, is a physiological compound for decreasing DP; and (iii) reduction of DP induced by α-linolenic acid is sufficient for significantly enhancing the cytoplasmic entry of penetratin.

The fact that the DP is localized in the membrane makes its measurement difficult, especially in living cells. DP of living cells can be determined with voltage-sensitive fluorophores. The application of these dyes is based on electrochromism, i.e., changes in their spectral properties in response to the local electric field. The excitation spectrum of di-8-ANEPPS is specifically sensitive to DP due to the localization of its chromophore group in the interfacial region (Gross et al., 1994; Clarke and Kane, 1997; Haldar et al., 2012; Kovacs et al., 2016, 2017). Therefore, excitation ratiometric methods can examine living cells in spectrofluorometry or microscopy. However, the former cannot give information about individual cells and includes data of dead cells, while the latter is not suitable for high-throughput techniques. A flow cytometric assay combines the beneficial properties of both approaches, but a dye shifting its emission spectrum in a DP-sensitive manner is preferred since it could be detected by simultaneous measurement of fluorescence intensities in two spectral ranges. Since di-8-ANEPPS was found to be inappropriate for such a method (Vitha and Clarke, 2007), we used F66, a 3-hydrixyflavone derivative, exhibiting DP-dependent changes in its emission spectrum due to excited-state intramolecular proton transfer (ESIPT). The interconversion and equilibrium between two excited states, the normal (N^∗^) and the tautomer (T^∗^) states, are largely modulated by DP leading to changes in the emission spectrum (Klymchenko et al., 2003; Shynkar et al., 2005; Darwich et al., 2013; Kovacs et al., 2017).

In the present study, we described and validated an emission ratiometric flow cytometry assay to determine alterations in the magnitude of DP. The reliability of the described method is supported by several lines of evidence: (i) 6KC and phloretin, two compounds most widely used to increase and decrease DP, respectively (Gross et al., 1994; Clarke and Kane, 1997; Kovacs et al., 2016, 2017), altered the emission characteristics of F66 measured by flow cytometry in two different cell lines as expected; (ii) three different sterols, 6KC, cholesterol and 7DHC, modified the flow cytometric emission ratio of F66 in accordance with their reported effects on DP; and (iii) the correlation coefficient between the intensity ratio of di-8-ANEPPS excited at two different wavelengths and the intensity ratio of F66 detected in two different wavelength ranges was close to one. Although all of the applied sterols were previously shown to increase DP, the extents of their effect are different, which may result from differences between their molecular structure, intrinsic dipole moment, localization in the membrane and their effects on water penetration and on the dielectric constant of the membrane. As a result, 6KC elevates DP more extensively than cholesterol (Simon et al., 1992; Smondyrev and Berkowitz, 2001; Starke-Peterkovic et al., 2006), while 7DHC induces much smaller changes in its magnitude (Haldar et al., 2012).

Having established the reliability of the new flow cytometric DP-measuring approach, we tested the effects of fatty acids on DP. We showed that ω-3 polyunsaturated ALA significantly decreased DP, while saturated SA and, to our surprise, ω-6 polyunsaturated GLA exerted an opposite effect as they slightly increased it. In addition, ALA counteracted, while SA and GLA slightly enhanced the DP elevating effect of cholesterol. ω-3 and ω-6 PUFAs are generally thought to exert quantitatively similar effects on membrane biophysical parameters, such as increased membrane fluidity (Calder et al., 1994; Leifert et al., 1999; Rajamoorthi et al., 2005), higher degree of hydration (Huster et al., 1997), decreased thickness and increased bending elasticity of lipid bilayers (Rawicz et al., 2000; Rajamoorthi et al., 2005). Similarly, both ω-3 and ω-6 PUFAs are characterized by beneficial effects on the prevalence of ischemic heart disease, which is partially attributed to their effects on serum lipid levels (Bang et al., 1980; Mensink et al., 2003; Harris et al., 2009; Del Gobbo et al., 2016; O’Mahoney et al., 2018; Li et al., 2020; Watanabe and Tatsuno, 2020). On the other hand, there are reports suggesting that ω-3 and ω-6 PUFAs are not created equal in all respects since some studies reported that ω-6, but not ω-3 PUFAs might in fact increase the risk of cardiovascular disorders (Simopoulos, 2008; Ramsden et al., 2013; Hamley, 2017; Nishizaki et al., 2020), while ω-3 and ω-6 PUFAs were generally shown to exert opposing effects on the incidence of malignant tumors (Cockbain et al., 2012; de Lorgeril and Salen, 2012; Fabian et al., 2015) with the former selectively inducing the apoptosis of tumor cells (D’Eliseo and Velotti, 2016). Although the aforementioned contrasting consequences of ω-6 and ω-3 PUFAs are generally thought to be mainly mediated by their soluble derivatives acting on signaling pathways and gene expression, several recent studies showed that changes in membrane biophysical parameters induced by these fatty acids can contribute to their effects on transmembrane proteins (Bruno et al., 2007; Caires et al., 2017; Romero et al., 2019). Since DP is closely linked to membrane structure, our results revealing different effects of ω-3 and ω-6 PUFAs on the DP suggest that these fatty acids exert slightly different effects on the biophysical properties of the membrane. Due to their unsaturated nature both of them “loosen” the hydrophobic core of the membrane (Calder et al., 1994; Leifert et al., 1999; Rajamoorthi et al., 2005), but differences in their conformation suggest that they have distinct effects on the lipid-water interface. The hydrophobic hydrocarbon chain of ALA (Pubchem ID: 5280934) folds back toward the hydrophilic head group very significantly, while the same tendency of GLA (Pubchem ID: 5280933) is much less pronounced. The tendency of the hydrophobic chain in ALA to approach the lipid-water interface has also been demonstrated by molecular dynamics simulations (Gawrisch et al., 2008). Therefore, we propose that the high probability of the hydrophobic hydrocarbon chain of ALA to be close to the lipid-water interface subverts the orderly arrangement of lipid head groups and interfacial water molecules leading to reduced DP. The lower tendency of the hydrophobic part of GLA to approach the lipid-water interface abolishes its DP-reducing effect. Consistent with our hypothesis, a recent MD simulation study demonstrated that besides dose-dependently inducing lateral expansion, thinning and decreased order in model bilayers, membrane incorporation of ω-3 PUFAs led to a significantly increased entry of water molecules into the hydrophobic regions represented by reduced distances between water-lipid interfaces, which were accompanied by a significantly altered cholesterol distribution. In the absence of ω-3 PUFAs, cholesterol molecules were mainly localized just below the phospholipid headgroups with their hydrated hydroxyl groups participating in a hydrogen bond with glycerol backbones of phospholipids. On the contrary, in the presence of ω-3 PUFAs, cholesterol is distributed in a more diffuse manner throughout the hydrophobic core of the bilayer resulting in reduced ordering, which might be associated with its hydroxyl group being hydrated even in deeper membrane regions due to increased water permeation (Ayee et al., 2020). Since ω-6 PUFAs are expected to localize differently in the membrane based on their slightly different molecular structures, these fatty acids might affect cholesterol and water distribution in an altered manner. This hypothesis could be confirmed by MD simulations revealing the intramembrane molecular organization of ω-6 PUFAs.

In our experiments, we tested the potential contribution of parameters other than DP to alterations in the emission ratio of F66 induced by sterols and fatty acids. It was shown previously that changes in the membrane during apoptosis may significantly alter spectral characteristics of F66 (Darwich et al., 2013). However, we could rule out this confounding effect since treatment conditions did not result in any significant change in cell viability (Figure 4A). Given the intimate relationship between DP, membrane fluidity and hydration (O’Shea, 2003, 2005; Wang, 2012; Zakany et al., 2020), we also examined the correlation between alterations in these parameters induced by the lipids used in our measurements. While the observed changes in membrane fluidity were in agreement with previously published data (Leifert et al., 1999; Chattopadhyay et al., 2007; Shrivastava et al., 2008), they did not show significant correlations with the determined F66 emission ratios arguing against a large contribution of membrane fluidity to changes induced in F66 spectrum (Figure 5). In these experiments, we measured the steady-state fluorescence anisotropy of TMA-DPH since this parameter reports on the fluidity in the membrane close to the lipid-water interface, i.e., the region of the DP, as opposed to its parent compound, DPH, whose signal averages the entire depth of the hydrophobic membrane interior (Prendergast et al., 1981; do Canto et al., 2016). Although the steady-state fluorescence anisotropy of TMA-DPH might be influenced by several factors related to dynamic and static aspects of fluidity, and therefore linking it to membrane order or fluidity is problematic, depolarization of TMA-DPH fluorescence does indeed correlate with membrane disorder (Engel and Prendergast, 1981; do Canto et al., 2016). Furthermore, TMA-DPH shows relatively slow internalization (Kubina et al., 1986), therefore its fluorescence originates almost exclusively from the cell membrane, similarly to F66 that we demonstrated previously to remain in the plasma membrane of cells under our experimental conditions (Kovacs et al., 2017). On the other hand, significant positive correlations were found between PY3174 generalized polarization and F66 emission ratios (Figure 5), which must be due to the proposed strong relationship between the organization of membrane-associated water molecules and the magnitude of DP (Simon et al., 1992; Smondyrev and Berkowitz, 2001; O’Shea, 2003, 2005; Starke-Peterkovic et al., 2006; Wang, 2012; Zakany et al., 2020).

One of the most widely studied cellular functions influenced by the magnitude of DP is the cellular binding and uptake of certain substances including drugs (Asawakarn et al., 2001), β-amyloid (Hertel et al., 1997) and other peptides (Cladera and O’Shea, 1998; Zhan and Lazaridis, 2012). We have also shown recently that lowering DP with phloretin or atorvastatin resulted in increased cellular uptake and, in particular, endo-lysosomal escape of penetratin, a cationic cell-penetrating peptide (Batta et al., 2020). Although cell-penetrating peptides are promising therapeutic tools for delivering cell-impermeable agents (Guidotti et al., 2017), their clinical potential is limited by low bioavailability (Wang et al., 2014). Therefore, any treatment enhancing their capability to reach the cytosol is of potential medical relevance. Both mechanisms of cellular entry of cell-penetrating peptides, direct plasma membrane translocation (Guidotti et al., 2017) and endocytosis followed by endo-lysosomal release (Futaki, 2006), involve electrostatic interactions with the membrane (Thoren et al., 2004; Poon and Gariepy, 2007). Therefore, both pathways might be modified by the intramembrane electric field associated with DP. Consistent with this hypothesis we found that the endo-lysosomal escape of penetratin was significantly enhanced by ω-3 polyunsaturated ALA and the concomitant decrease in DP. Since ALA-induced increased endo-lysosomal release of penetratin increases its bioavailability in the cytosol, the therapeutic effectiveness of drugs conjugated to penetratin is expected to increase. On the other hand, fully saturated SA or ω-6 polyunsaturated GLA, which rather elevate DP, failed to influence cytoplasmic penetratin entry (Figure 7). These findings corroborate the effects of DP on the uptake of cell-penetrating peptides and emphasize the physiological importance of ω-3 PUFA mediated decreases in DP. In our experiments we applied flow cytometry with fluorophore-conjugated penetratins to quantify DP-mediated effects on the cellular uptake and endo-lysosomal release of these cell-penetrating peptides. While the presence of fluorescent labels might influence the membrane binding of these peptides (Christensen et al., 2019), and this can be overcome by the use of label-free techniques such as NMR spectroscopy (Christensen et al., 2019), measurement of intrinsic fluorescence of these molecules (Jobin and Alves, 2016) or mass spectrometry (Burlina et al., 2005), fluorescence-based approaches still remain the most commonly used techniques due to their unique advantages, including suitability for high-throughput examinations, easy-to-use application and ability to estimate penetratin levels in various intracellular compartments and to discriminate between live and dead cells. The latter is particularly crucial in these experiments in light of our recent observations that damaged cells are characterized by larger instantaneous binding of penetratin with much smaller increases in its time-dependent uptake when compared with intact cells (Batta et al., 2020). Nevertheless, care has to be taken in the interpretation of results obtained with methods applying fluorescently labeled penetratins. The potential biological relevance of our findings is also supported by the concentrations applied in the treatments. Membrane cholesterol levels achieved by the treatments applied were comparable to those found in patients with hypercholesterolemia (Somodi et al., 2013). Since loading of cells with 7DHC and cholesterol is equally efficient (Singh et al., 2007), the levels of 7DHC and cholesterol of cells treated with the MβCD complexes of these compounds is expected to be comparable. The fact that membrane 7DHC levels of lymphocytes obtained from Smith-Lemli-Opitz syndrome patients were comparable to cholesterol levels of lymphocytes from control individuals (Balajthy et al., 2016) argues for the relevance of the 7DHC concentrations applied. Fatty acid concentrations used in our experiments were also in the range of serum levels usually obtained in studies examining effects of dietary supplementation of ω-3 PUFAs (Conquer and Holub, 1998; Kuriki et al., 2002).

Potential limitations of the DP determination method described here might arise from the fact that being a flow cytometry-based ensemble approach it might mask heterogeneous changes in the magnitude of DP hidden within the determined average values. However, as opposed to the conventional spectrofluorometric assays based on di-8-ANEPPS, it provides the possibility to identify subpopulations of cells based on combinations of forward and side scatter parameters and fluorescence intensities of various labels. Furthermore, besides solely focusing on average values, other statistical parameters such as SD or SEM should be taken into account since these might carry relevant biological information. For example, a largely increased variance might refer to remarkable heterogeneity in the examined cell population. On the other hand, as we demonstrated previously (Kovacs et al., 2017), lateral heterogeneity of DP can be a functionally relevant property of biological membranes, which cannot be easily investigated with a flow cytometry-based approach. Nevertheless, the emission ratio of the F66 fluorophore can also be used to examine the spatial heterogeneity of DP in the cell membrane in confocal microscopy, as we showed recently (Kovacs et al., 2017). Based on these considerations, a multimodal approach applying both flow cytometry and confocal microscopy could provide the most detailed information about changes in the magnitude of DP in response to alterations in membrane composition.

In conclusion, in this study we have developed and optimized a flow cytometric DP measurement technique suitable for high-throughput examination of large quantities of living cells. Furthermore, we have identified ω-3 polyunsaturated ALA as a physiological tool to lower the magnitude of DP and demonstrated the biological relevance of this effect by showing enhanced cellular uptake and endo-lysosomal escape of a cell-penetrating peptide. Our novel method and the applicability of a physiological DP lowering agent could provide a boost for the examination of DP, an enigmatic and proposedly substantial membrane biophysical parameter.

## Materials and Methods

### Cell Culture and Treatments to Induce Changes in the Magnitude of Dipole Potential

The human acute monocytic leukemia-derived cell line THP-1 and the human Epstein-Barr virus (EBV) transformed lymphoblastoid cell line JY were obtained from the American Type Culture Collection (Manassas, VA, United States) and cultivated according to their specifications. To induce changes in the magnitude of DP, cells were treated with phloretin (3-(4-hydroxyphenyl)-1-(2,4,6-trihydroxyphenyl) propan-1-one) (Sigma Aldrich, St. Louis, MO, United States) or 6-ketocholestanol (3β-hydroxy-5α-cholestan-6-one, 6KC) (Sigma Aldrich) at various concentrations between 20 and 200 μM for 10 min at room temperature in the presence of 0.05% (v/v) Pluronic F-127. Alternatively, cells were loaded with 6KC, cholesterol (Sigma-Aldrich) or 7-dehydrocholesterol (7DHC) (Sigma-Aldrich) using custom synthetized sterol-methyl-beta-cyclodextrin (MβCD) complexes (CycloLab Cyclodextrin R&D Laboratory, Budapest, Hungary) at various sterol concentrations ranging from 12.5 to 200 μM for 60 min at room temperature. As controls, cells were treated with the corresponding amounts of native MβCD complexes not containing sterols. To study the effects of fatty acids, cells were incubated for 48 h in medium supplemented with saturated stearic acid (SA, Sigma Aldrich), ω-3 polyunsaturated α-linolenic acid (ALA, Sigma Aldrich) or ω-6 polyunsaturated γ-linolenic acid (GLA, Sigma Aldrich) at various concentrations ranging from 12.5 to 50 μM.

### Membrane Dipole Potential Measurement With Spectrofluorometry

Following treatment with different concentrations of DP-modifying agents, cells were labeled with di-8-ANEPPS (4-(2-[6-(dioctylamino)-2-naphthalenyl]ethenyl)-1-(3-sulfopropyl) pyridinium inner salt, Thermo Fisher Scientific, Waltham, MA, United States) at a final concentration of 2 μM on ice for 20 min. After washing, cells were kept on ice and samples were subsequently warmed to 37°C right before measurements carried out with a Fluorolog-3 spectrofluorometer (Horiba Jobin Yvon, Edison, NJ, United States). During measurements, the cuvette holder was kept at 37°C using a circulating water bath. Excitation spectra were recorded between 380 and 550 nm with the detected emission wavelength set to 660 nm in order to minimize effects of possible changes in membrane fluidity (Clarke and Kane, 1997). The ratio of fluorescence intensities integrated between excitation wavelengths 410–440 nm and 490–520 nm was calculated for each sample, which positively correlates with the magnitude of DP (Clarke and Kane, 1997; Kovacs et al., 2017; Batta et al., 2020).

### Measuring the Dipole Potential Using Flow Cytometry

Cells treated with different DP-modifying agents were incubated in the presence of 10 nM F66 (N-[3-(40-di hexylamino-3-hydroxy-flavonyl-6-oxy)-propyl] N,N-dimethyl-N-(3-sulfopropyl)-ammonium inner salt, a kind gift from Andrey Klymchenko, Université de Strasbourg, Strasbourg, France) for 20 min on ice. After washing, the fluorescence intensities of cells were determined with a FACS Aria III flow cytometer (Becton Dickinson, Mountain View, CA, United States) at 37°C. The dye was excited at 405 nm and its emission was measured using band pass filters of 480/20 nm and 585/42 nm, corresponding to the normal (N^∗^) and tautomer (T^∗^) excited states of the flavone chromophore, respectively. Data analysis was carried out in FCS Express (De Novo Software, Los Angeles, CA, United States). The ratio of emitted intensities in the two wavelength ranges (N^∗^/T^∗^) was determined for each cell and the average value was calculated for each sample from the data of living cells gated on FSC-SSC dot plots. The value of N^∗^/T^∗^ ratio negatively correlates with the magnitude of DP (Kovacs et al., 2017).

### Measurement of Cell Viability

Treated or control THP-1 or JY cells seeded into 24-well plates were labeled with Sytox Green Dead Cell Stain (Thermo Fisher Scientific) and AlexaFluor647-conjugated annexin V (Thermo Fisher Scientific) at dilutions of 1:1,000 and 1:20, respectively, in annexin binding buffer for 15 min at room temperature. Fluorescence intensities of individual cells were subsequently measured using a NovoCyte 3000RYB flow cytometer (ACEA Biosciences, San Diego, CA, United States). Sytox Green and AlexaFluor647 fluorophores were excited at 488 and 640 nm, respectively, and emitted intensities were measured using 530/30 and 660/20 emission filters, respectively. During data analysis, the fraction of Sytox Green and annexin V negative living cells was calculated for each sample using FCS Express.

### Measurement of Membrane Fluidity With Spectrofluorometry

4′-(trimethylammonio)-diphenylhexatriene (TMA-DPH) was purchased from Sigma-Aldrich, dissolved in tetrahydrofuran and used for determination of membrane fluidity as described previously (Batta et al., 2018). Briefly, control and treated cells were washed and resuspended in Hank’s buffer followed by labeling with 10 μM TMA-DPH for 20 min at room temperature. Fluorescence intensities were measured without washing using a Fluorolog-3 spectrofluorometer (Horiba Jobin Yvon, Edison, NJ, United States) with the temperature of the cuvette holder adjusted to 37°C by a circulating water bath. The fluorescence anisotropy (*r*) of TMA-DPH was determined in the L-format after excitation at 352 nm and measurement of fluorescence intensities at 430 nm according to the formula:

(1) $$r=\frac{I_{vv}-GI_{vh}}{I_{vv}+2GI_{vh}}$$

where *I*_*vv*_ and *I*_*vh*_ are the vertical and horizontal components, respectively, of the fluorescence excited by vertically polarized light, and *G* is an instrument-specific correction factor characterizing the different sensitivity of the detection system for vertically and horizontally polarized light:

(2) $$G=\frac{I_{hv}}{I_{hh}}$$

where *I*_*hv*_ and *I*_*hh*_ are the vertical and horizontal components, respectively, of the fluorescence excited by horizontally polarized light.

### Measurement of Membrane Hydration With Fluorescence Microscopy

The PY3174 fluorophore capable of providing information about the hydration of the cell membrane via an emission ratiometric fluorescence microscopy assay was a kind gift from Leslie M. Loew (University of Connecticut, CT, United States) (Kwiatek et al., 2013). For these measurements, cells were labeled with 10 μM PY3174 for 20 min at room temperature. After staining, cells were placed onto an 8-well chambered coverglass and images were taken at the midplane of cells using an LSM880 confocal laser-scanning microscope (Carl Zeiss AG, Jena, Germany). PY3174 was excited at 488 nm and emitted intensities were measured in two wavelength ranges between 500 and 540 nm (I_*blue*_) and 630 and 735 nm (I_*red*_). During processing, segmentation of images into membrane and non-membrane pixels was carried out with the manually seeded watershed algorithm using a custom-written MATLAB program as described previously (Kovacs et al., 2017). The average value of general polarization correlating with the degree of membrane compactness (Kwiatek et al., 2013) was calculated from the data of cell membrane pixels after background subtraction using

(3) $$GP=\frac{I_{blue}-I_{red}}{I_{blue}+I_{red}}.$$

### Synthesis and Fluorescence Labeling of Penetratin

Penetratin (RQIKIWFQNRRMKWKK-amide, molecular weight 2245.75 g/mol) was synthesized manually on TentaGel R RAM (Rapp Polymere, Tübingen, Germany), a low crosslinked polystyrene PEG copolymer resin by the solid-phase method of Merrifield with standard Fmoc chemistry, as described in detail in our recent study (Batta et al., 2020). After coupling the last arginine, one half of the penetratin was reacted with AFDye532 N-hydroxysuccinimide ester (molecular weight 723.77 g/mol, Fluoroprobes, Scottsdale, AZ, United States), while the other half with 5(6)-carboxynaphthofluorescein N-succinimidyl ester (molecular weight 573.51 g/mol, Darmstadt, Germany). Completion of the coupling was assessed by Kaiser test, which was followed by deprotection and release from the resin. After filtration and precipitation with cold diethyl ether, the crude products were purified by preparative reversed-phase HPLC (JASCO, Victoria, BC, Canada) on a C18 column and lyophilized. The purity of the products (>95%) was assessed by reversed-phase HPLC (JASCO) equipped with an analytical C18 column. The presence of labeled peptides was validated with Bruker electrospray ionization mass spectrometry that showed the molecular mass of the (M+H)^+^ form of AFDye532- and NF-labeled penetratin as 2983.548 and 2832.5, respectively. These values well correlated with the predicted masses of the labeled peptides (2983.59 and 2833.33, respectively).

### Flow Cytometric Measurement of Penetratin Uptake and Endo-Lysosomal Escape

Total cellular penetratin uptake and endo-lysosomal escape was determined as described previously (Batta et al., 2020). Briefly, control THP-1 or JY cells and those treated with 50 μM ALA, GLA or SA for 48 h at 37°C were incubated in the continuous presence of 5 μM AFDye532-penetratin, 5 μM NF-penetratin and 0.25 μg/ml DAPI at 37°C in the thermostated sample holder of a FACS Aria III flow cytometer. The fluorescence intensity of DAPI was measured using an excitation at 405 nm and an emission filter 450/20 nm. AFDye532 and NF were excited at 488 and 561 nm, respectively, and their fluorescence was determined using emission filters 530/30 and 670/14 nm, respectively. Measurements started immediately after addition of DAPI and penetratin and continued for 20 min at 37°C. Experimental data were analyzed first in FCS Express. Time-correlated fluorescence intensity values of DAPI-negative living cells were exported after spectral compensation and the moving averages of samples were calculated with a window size of 10 s. While the AFDye532 is not sensitive to pH and shows total cellular penetratin uptake, the intensity of NF is quenched at acidic pH, thus the ratio of NF-penetratin to AFDye532-penetratin signal refers to the endo-lysosomal escape (Qian et al., 2015). During analysis, fluorescence intensity values were first normalized to the average intensity in the first time window, which was followed by calculation of the fluorescence ratio from the normalized values.

### Statistical Analysis

Measured data are generally represented as mean ± SD obtained from at least five different experiments (*n*). In measurements carried out with a flow cytometer, at least 20,000 cells per sample were analyzed in each independent experiment. In experiments with sterol-MβCD complexes, values were normalized to samples treated with equivalent amounts of native MβCD, while in the cases of fatty acids to the values obtained with ethanol controls. *P* values were calculated based on ANOVA followed by Tukey’s HSD test. Differences were considered significant (^∗^) when *p* < 0.05.

## Data Availability Statement

The raw data supporting the conclusions of this article will be made available by the authors, without undue reservation.

## Author Contributions

FZ: conceptualization, formal analysis, investigation, methodology, visualization, and writing – original draft preparation. MS: investigation. GB: methodology and visualization. LK: resources. IM: funding and resources. PF: investigation and writing – review and editing. ZV and GP: funding and writing – review and editing. PN: formal analysis, funding, methodology, supervision, and writing – review and editing. TK: conceptualization, formal analysis, methodology, project administration, visualization, writing – original draft preparation, and writing – review and editing. All authors contributed to the article and approved the submitted version.

## Conflict of Interest

The authors declare that the research was conducted in the absence of any commercial or financial relationships that could be construed as a potential conflict of interest.

## Abbreviations

6KC: 6-ketocholestanol
7DHC: 7-dehydrocholesterol
ALA: α-linolenic acid
DP: dipole potential
ESIPT: excited-state intramolecular proton transfer
GLA: γ-linolenic acid
M β CD: methyl-beta-cyclodextrin
N^∗^: normal excited state
PUFA: polyunsaturated fatty acid
R_*em, F66*_: emission ratio of the F66 fluorophore
R_*exc, di–8–ANEPPS*_: excitation ratio of the di-8-ANEPPS fluorophore
SA: stearic acid
T^∗^: tautomer excited state.
