# Supplementary material for: An ω-3, but Not an ω-6 Polyunsaturated Fatty Acid Decreases Membrane Dipole Potential and Stimulates Endo-Lysosomal Escape of Penetratin
Source: Front Cell Dev Biol. 2021 Apr 12;9:647300. doi: 10.3389/fcell.2021.647300 (PMC8074792; doi:10.3389/fcell.2021.647300)
Supplement: Supplementary file 1 [file Data_Sheet_1.PDF]

Supplementary Material

1 Supplementary Methods

Measurement of hydration of lysosomal membranes with fluorescence microscopy

The PY3174 fluorophore capable of providing information about the hydration of the cell membrane via an emission ratiometric fluorescence microscopy assay was a kind gift from Leslie M. Loew (University of Connecticut, CT) (1). For these measurements, cells were placed onto an 8-well chambered coverglass and labeled with 100 nM LysoTracker Deep Red (Thermo Fisher Scientific, Waltham, MA) for visualization of lysosomes and 10 µM PY3174 for 2 hours at 37°C to stimulate the endocytosis of the environment-sensitive fluorophore. After staining, images were taken at the midplane of cells using an LSM880 confocal laser-scanning microscope (Carl Zeiss AG, Jena, Germany). PY3174 was excited at 488 nm and emitted intensities were measured in two wavelength ranges between 500 and 540 nm (I_{blue}) and 650 and 735 nm (I_{red}), while LysoTracker Deep Red was excited at 633 nm and emission was detected between 650 and 735 nm. During processing, pixels corresponding to lysosomal membranes were identified based on the intensity of LysoTracker Deep Red using a custom-written algorithm under MATLAB. The average value of generalized polarization (GP) correlating with the degree of membrane hydration (1) was calculated from the data of lysosomal membrane pixels after background subtraction using

$$GP = \frac{I_{blue} - I_{red}}{I_{blue} + I_{red}} \quad (1).$$

2 Supplementary Figure

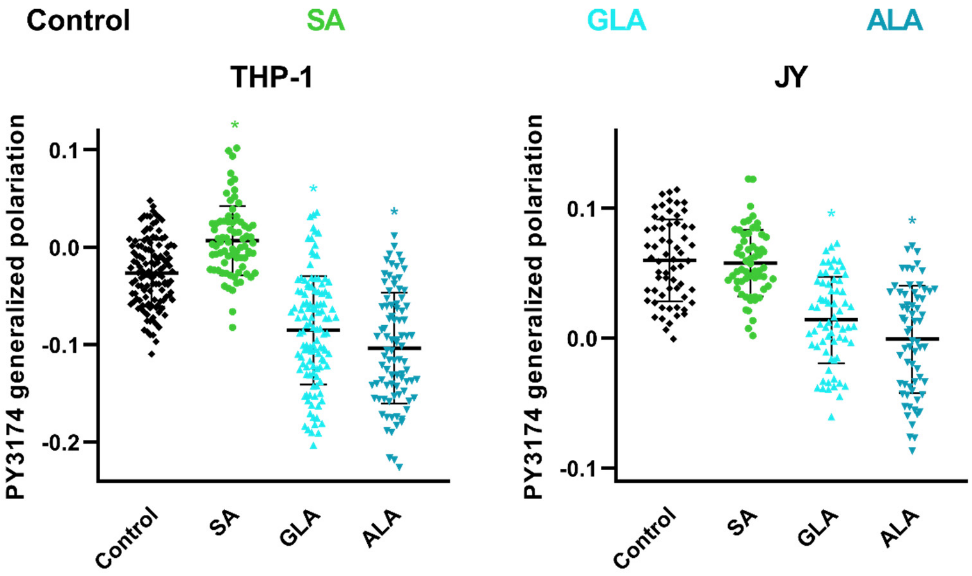

Supplementary Figure 1. Effects of fatty acids on the hydration of lysosomal membranes. (A) THP-1 or JY cells were treated with 50 µM ALA (blue), GLA (cyan) or SA (green) for 48 h, and subsequently labeled with LysoTracker Deep Red and PY3174 for 2 h to stimulate endocytosis of the environment-sensitive fluorophore, which was followed by determination of the generalized polarization of the dye localized in lysosomal membranes using confocal microscopy and quantitative image analysis. Mean generalized polarization values of 70-110 individual cells of normal morphology obtained in five independent experiments and their average values (± SD) are plotted for the different treatments. Asterisks (*) indicate significant differences compared to control samples ($p<0.05$, ANOVA followed by Tukey's HSD test).

3 Supplementary Reference

1. Kwiatek JM, Owen DM, Abu-Siniyeh A, Yan P, Loew LM, Gaus K. Characterization of a new series of fluorescent probes for imaging membrane order. *PLoS One* (2013) 8(2):e52960. doi: 10.1371/journal.pone.0052960. PubMed PMID: 23390489; PubMed Central PMCID: PMCPMC3563652.
